# A novel small-molecule PROTAC selectively promotes tau clearance to improve cognitive functions in Alzheimer-like models

**DOI:** 10.7150/thno.55680

**Published:** 2021-03-11

**Authors:** Weijin Wang, Qiuzhi Zhou, Tao Jiang, Shihong Li, Jinwang Ye, Jie Zheng, Xin Wang, Yanchao Liu, Minmin Deng, Dan Ke, Qun Wang, Yipeng Wang, Jian-Zhi Wang

**Affiliations:** 1 Department of Pathophysiology, School of Basic Medicine, Key Laboratory of Education Ministry of China/Hubei Province for Neurological Disorders, Tongji Medical College, Huazhong University of Science and Technology, Wuhan 430030, China.; 2 Neurosmart Therapeutics Co., Ltd., Room 5013, Unit 1, Buiilding 7, Basheng road 160, Shanghai 200131, China.; 3 Co-innovation Center of Neuroregeneration, Nantong University, Nantong 226000, China.

**Keywords:** tau, proteolysis targeting chimeras, C004019, Alzheimer's disease, tauopathies

## Abstract

Intracellular accumulation of tau is a hallmark pathology in Alzheimer disease (AD) and the related tauopathies, thus targeting tau could be promising for drug development. Proteolysis Targeting Chimera (PROTAC) is a novel drug discovery strategy for selective protein degradation from within cells.

**Methods:** A novel small-molecule PROTAC, named as C004019 with a molecular mass of 1,035.29 dalton, was designed to simultaneously recruite tau and E3-ligase (Vhl) and thus to selectively enhance ubiquitination and proteolysis of tau proteins. Western blotting, immunofluoresence and immunohistochemical staining were employed to verify the effects of C004019 in cell models (HEK293 and SH-SY5Y) and mouse models (hTau-transgenic and 3xTg-AD), respectively. The cognitive capacity of the mice was assessed by a suite of behavior experiments. Electrophysiology and Golgi staining were used to evaluate the synaptic plasticity.

**Results:** C004019 induced a robust tau clearance *via* promoting its ubiquitination-proteasome-dependent proteolysis in HEK293 cells with stable or transient overexpression of human tau (hTau), and in SH-SY5Y that constitutively overexpress hTau. Furthermore, intracerebral ventricular infusion of C004019 induced a robust tau clearance *in vivo*. Most importantly, both single-dose and multiple-doses (once per 6 days for a total 5 times) subcutaneous administration of C004019 remarkably decreased tau levels in the brains of wild-type, hTau-transgenic and 3xTg-AD mice with improvement of synaptic and cognitive functions.

**Conclusions:** The PROTAC (C004019) created in the current study can selectively and efficiently promote tau clearance both* in vitro* and *in vivo*, which provides a promising drug candidate for AD and the related tauopathies.

## Introduction

Alzheimer disease (AD) is the most common neurodegenerative disorder characterized clinically by deterioration of the cognitive functions and progressive loss of the memory 1. With the increasing of the longevity, the increased prevalence rate of AD has become a major health concern and financial burden for the society. It is estimated that 35 million people worldwide are suffering AD 2, and this figure will reach at least 100 million by 2050 3. As the pathogenesis of AD is still unclear, there is currently no effective therapy or drug for this devastating disease, highlighting an urgent unmet need for drug development.

Intracellular accumulation of the hyperphosphorylated tau (p-tau) and the extracellular precipitation of amyloid-β (Aβ) are hallmark pathologies in the AD brains 4, 5. In the past decades, AD drug development has been primarily focused on Aβ. Accordingly, various Aβ-targeting therapies have been developed and tested, such as reducing Aβ production by β-/γ-secretase inhibitors 6, 7, removing Aβ by promoting its degradation or antibodies 8, 9. Unfortunately, the clinical effects of these Aβ-targeting strategies have been disappointing, to date. Recently, the therapies against tau proteins or the tau-related pathways have been proposed, these include reducing tau expression with small interfering RNA (siRNA) 10 or antisense oligonucleotides (ASOs) 10; modulating tau post-translational modifications by regulating related enzymes activity, or tau aggregation by inhibitors 11, or tau degradation by autophagy-lysosome pathway (ALP) or ubiquitin-proteasome system (UPS) stimulators, or tau transmission by antibodies, or tau binding by microtubule stabilizers 12. For ablating previously undruggable proteins, an emerging strategy, known as targeted protein degraders or PROTACs (Proteolysis Targeting Chimeras), has recently caught attention for selective tau degradation 13-16.

Based on the principle of PROTAC, we designed a small molecule named C004019 in the present study. The two functional parts of C004019 respectively binds to tau proteins and an E3 ligase to reach a selective and efficient ubiquitination and the subsequent proteasome-mediated degradation of tau. By *in vitro* and *in vivo* testing, we found that C004019 could robustly promote tau clearance in multiple tau-overexpressing cell models *via* ubiquitination-proteasome pathway. Furthermore, both intracerebral and subcutaneous administration of C004019 could also remarkably reduce tau level in wild-type, human tau (hTau) transgenic and 3xTg-AD mouse models with simultaneous amelioration of synaptic and cognitive functions.

## Results

### C004019 induces tau clearance in different cell models *via* ubiquitin-proteasome system

**Figure 1:**
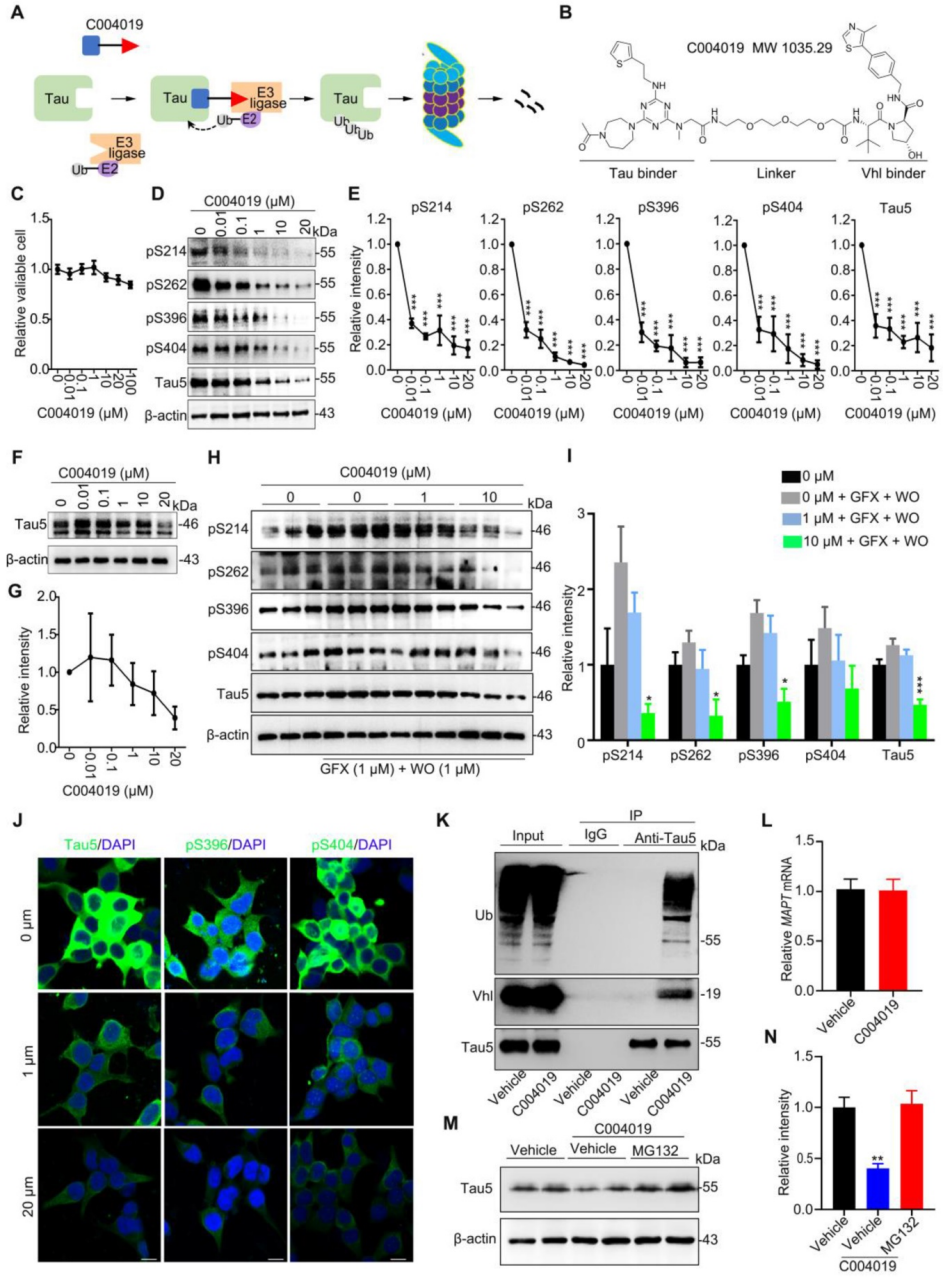
** C004019 induces tau clearance *via* promoting its ubiquitination and proteasome-dependent proteolysis *in vitro*. (A)** Working model of C004019. **(B)** The designed C004019 composed of a tau binder, a linker and a Vhl (E3 ligase) binder with a molecular mass of 1,035.29 Dalton. **(C)** Compared with the vehicle, C004019 (0.01-100 µM for 24 h) did not significantly affect cell viability measured by CCK-8 assay in HEK293-hTau cells. **(D-I)** C004019 induced a concentration-dependent clearance of total tau (Tau5) or p-tau at multiple-doses AD-related sites in HEK293-hTau cells (D-E) and SH-SY5Y cells (F-I) measured by Western blotting. (H-I) SH-SY5Y cells were simultaneously treated with C004019 (1 or 10 µM) and wortmainnin (WO, 1 µM) and GF-109203X (GFX, 1 µM) for 24 h to induce tau hyperphosphorylation, and then tau was measured by Western blotting. **(J)** C004019 induced tau reduction in HEK293-hTau cells measured by immunofluorescence staining (Scale bar, 10 µm). **(K)** Co-immunoprecipitation analysis showed interaction of tau with Vhl (E3 ligase) and the increased tau ubiquitination in HEK293-hTau cells treated with 20 µM C004019 for 6 h. **(L)** C004019 (20 µM for 24 h) did not affect mRNA level of tau in HEK293-hTau cells measured by q-PCR. **(M-N)** Inhibition of proteasome with MG132 (10 µM) abolished C004019-induced tau clearance measured by Western blotting. Data were expressed as mean ± SEM, (E) ***P < 0.001 vs. 0 µM. (I) *P < 0.05, ***P < 0.001 vs. 0 µM + GFX + WO. (N) **P < 0.01 vs. Vehicle. Data in (C, E, G, I, N) were analyzed by one-way ANOVA. Data in (L) was analyzed by Student's t-test.

To achieve a selective and efficient proteolysis of tau, we designed a PROTAC, named as C004019 with a molecular mass of 1,035.29 dalton (Figure 1A-B). The chimera is composed of three parts including a tau binder, a linker and an E3 ligase recruiter or Vhl binder, in which the two functional parts respectively binds to tau proteins and E3 ligase to reach a selective and efficient ubiquitination and the subsequent proteasome-mediated proteolysis of tau (Figure 1A-B).

We first tested the toxicity of the molecule by CCK8 assay in HEK293 cells with stable expression of wild-type full-length human tau (termed HEK293-hTau). The results showed that application of C004019 at concentrations of 0.01 μM, 0.1 μM, 1 μM, 10 μM, 20 μM and 100 μM did not induce any significant change in cell viability (Figure 1C). These data indicate that C004019 has very low cell toxicity.

Then, we measured the effect of C004019 on tau clearance in different cell models, including HEK293-hTau cells (with stable hTau expression), HEK293 cells with transient expression of human tau (HEK293-3xFlag-hTau or HEK293-EGFP-hTau), and the human neuroblastoma SH-SY5Y cells which constitutively express human tau proteins. We observed that C004019 treatment induced a concentration-dependent reduction of total tau (Tau5) and tau phosphorylated (p-tau) at multiple AD-related sites in HEK293-hTau (Figure 1D-E) and SH-SY5Y (Figure 1F-I) cells detected by Western blotting. We also observed that C004019 (20 μM) could decrease phosphorylated tau in SH-SY5Y cells (Figure S1E), but the potency of C004019 appeared much weaker in SH-SY5Y cells than the HEK293-hTau cells. As the tau proteins in SH-SY5Y cells were endogenously expressed in physiological conditions while the tau proteins in HEK293-hTau cells were exognenously overexpressed and pathologically accumulated, it is suggested that C004019 is more efficient in removing the pathological tau proteins. This is potentially significant for C004019 to be an efficient drug with limited side effect.

As SH-SY5Y cells have very limited phosphor-tau level, we used wortmannin (WO) plus GF109203X (GFX) to promote tau phosphorylation and then measured the effects of C004019. Previous studies demonstrated that WO plus GFX could robustly activate glycogen synthase kinase-3β (GSK-3β), a kinase that promotes tau hyperphosphorylation at multiple AD-associated sites 17, 18. By simultaneously treated the SH-SY5Y cells with C004019 (1 μM or 10 μM) and WO (1 μM) plus GFX (1 μM), we observed that C004019 at 10 μM could efficiently decrease p-tau and total tau levels (Figure 1H-I). The C004019 concentration-dependent clearance of total and p-tau proteins was also detected by immunofluorescence staining in HEK293-hTau cells (Figure 1J), and by Western blotting in HEK293-EGFP-hTau cells (Figure S1A-B). To exclude the influence of tag-EGFP, we also detected in HEK293-3xFlag-hTau cells. Same results were observed (Figure S1C-D), which excluded any potential interference of tag-EGFP. Additionally, C004019 could efficiently remove tau proteins at a concentration as low as 0.01 μM with IC50 = 0.00785 μM in HEK293-hTau cells (Figure S1G). By treated the HEK293-hTau cells with different concentrations of tau binder (2 μM, 20 μM, and 200 μM) and 20 μM C004019, we observed that tau binder at high concentrations (20 μM and 200 μM) blocked the effect of C004019 in removing tau proteins measured by Western blotting (Figure S1F). C004019 did not significantly change the mRNA level of tau (Figure 1L). These data together suggested that C004019 could selectively decrease both total and the phosphorylated tau proteins* in vitro*.

To confirm whether C004019 could indeed form the expected ternary structure and thus promotes ubiquitination of tau, we measured the binding of tau with Vhl and the consequent changes of tau ubiquitination by co-immunoprecipitation in HEK293-hTau cells. The association of tau with Vhl was detected, and C004019 treatment significantly increased the ubiquitination level of tau (Figure 1K). The association of tau with Vhl and the consequently increased ubiquitination of tau was also detected in HEK293-EGFP-hTau cells (Figure S1H). Furthermore, the C004019-induced clearance of tau was abolished by simultaneous treatment of HEK293-hTau cells with MG132, a proteasome inhibitor (Figure 1M-N). These data suggest that C004019 can robustly reduce tau proteins *via* promoting tau ubiquitination and its subsequent proteolysis by proteasome *in vitro*.

### Intracerebroventricular administration of C004019 induces *in vivo* tau clearance in wild-type and 3xTg mice

**Figure 2:**
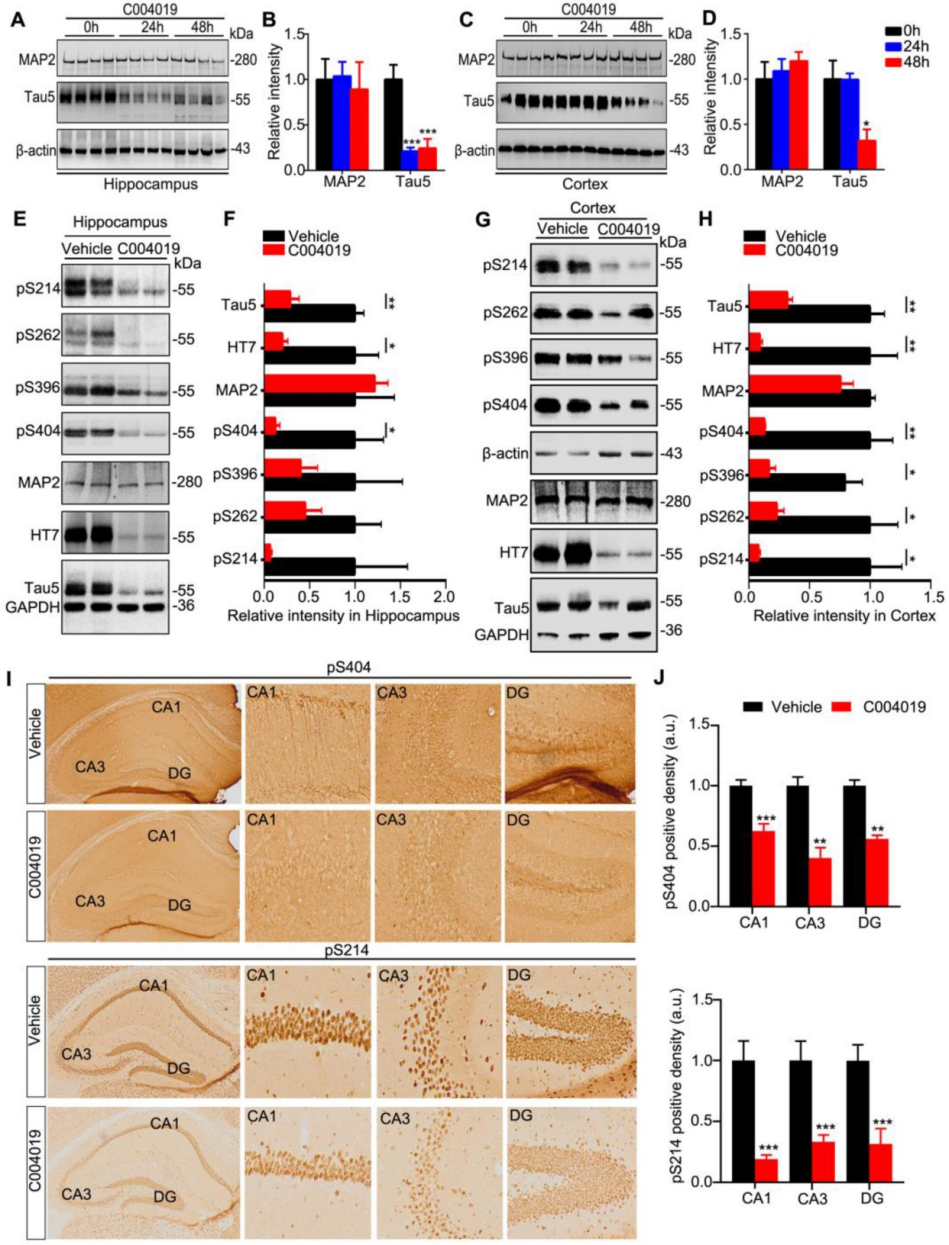
** Intracerebroventricular infusion of C004019 decreased tau in 4 m-old wild-type mice and 9.5 m-old 3xTg AD mice. (A-D)** Single-dose intracerebroventricular infusion of C004019 (5 µL, 200 µM) for 24 h and 48 h decreased tau level without affecting MAP2 in hippocampus (A-B) and cortex (C-D) in wild-type mice, more significantly in the hippocampus. β-actin was used as a loading control. **(E-H)** Intracerebroventricular infusion of C004019 (5 µL, 200 µM) in 9.5 m-old 3xTg-AD mice for 48 h significantly decreased total tau and the tau phosphorylated at multiple-doses AD-related sites in hippocampus (E-F) and cortex (G-H) measured by Western blotting. **(I-J)** C004019 decreased pS214 and pS404 tau in hippocampal CA1 and CA3 subsets measured by immunohistochemistry (Scale bar: 50 µm). GAPDH with same exposure in the same membrance was used as a loading control. Data were expressed as mean ± SEM, (B, D) *P < 0.05, ***P < 0.001 vs. 0 h. (F, H, J) *P < 0.05, **P < 0.01, ***P < 0.001vs. Vehicle. Data were analyzed by one-way ANOVA.

To explore whether C004019 could also promote tau clearance *in vivo*, we first infused C004019 (5 μL, 200 μM) into 4 m-old wild-type mice through lateral ventricle (posterior 0.22 mm, lateral 0.9 mm, and ventral 2.3 mm relative to bregma). After 24 h or 48 h, the changes of tau protein level in hippocampus and cortex were measured by Western blotting. The intracerebroventricular infusion of C004019 induced most remarkable hippocampal tau reduction at 24 h, and the reduction was still very significant at 48 h though it was slightly less than 24 h (Figure 2A-B). It is possible that the compound needs time for CSF penetration and circulation by *in vivo* administration, while a direct interaction of the compound with the* in vitro* cultured cell requires shorter time for the function. In the brain cortex, significant reduction of tau was only detected at 48 h but not 24 h (Figure 2C-D). These data suggested that C004019 could reduce tau proteins in the mouse brain after intracerebroventricular infusion, most efficiently in the hippocampus.

To explore the effect of C004019 on the pathological tau in AD mouse models, we infused C004019 (5 μL, 200 μM) into the lateral ventricles of 9.5 m-old 3xTg-AD mice which contained prominent tau pathology 19, 20. The results showed that intracerebroventricular administration of C004019 reduced total tau and the p-tau levels in hippocampus and cortex measured by Western blotting (Figure 2E-H) and immunohistochemistry (Figure 2I-J). To confirm the specificity of C004019, we measured the protein level of MAP2, another microtubule associated cytoskeletal protein. The results showed that C004019 did not change the level of MAP2 in both 4 m-old wild-type and 9.5 m-old 3xTg-AD mice (Figure 2A-H). These data confirmed that direct cerebroventricular administration of C004019 promotes a specifical and robust clearance of the pathological tau *in vivo*.

### Single-dose subcutaneous administration of C004019 induces tau clearance in the brain of wild-type mice

**Figure 3:**
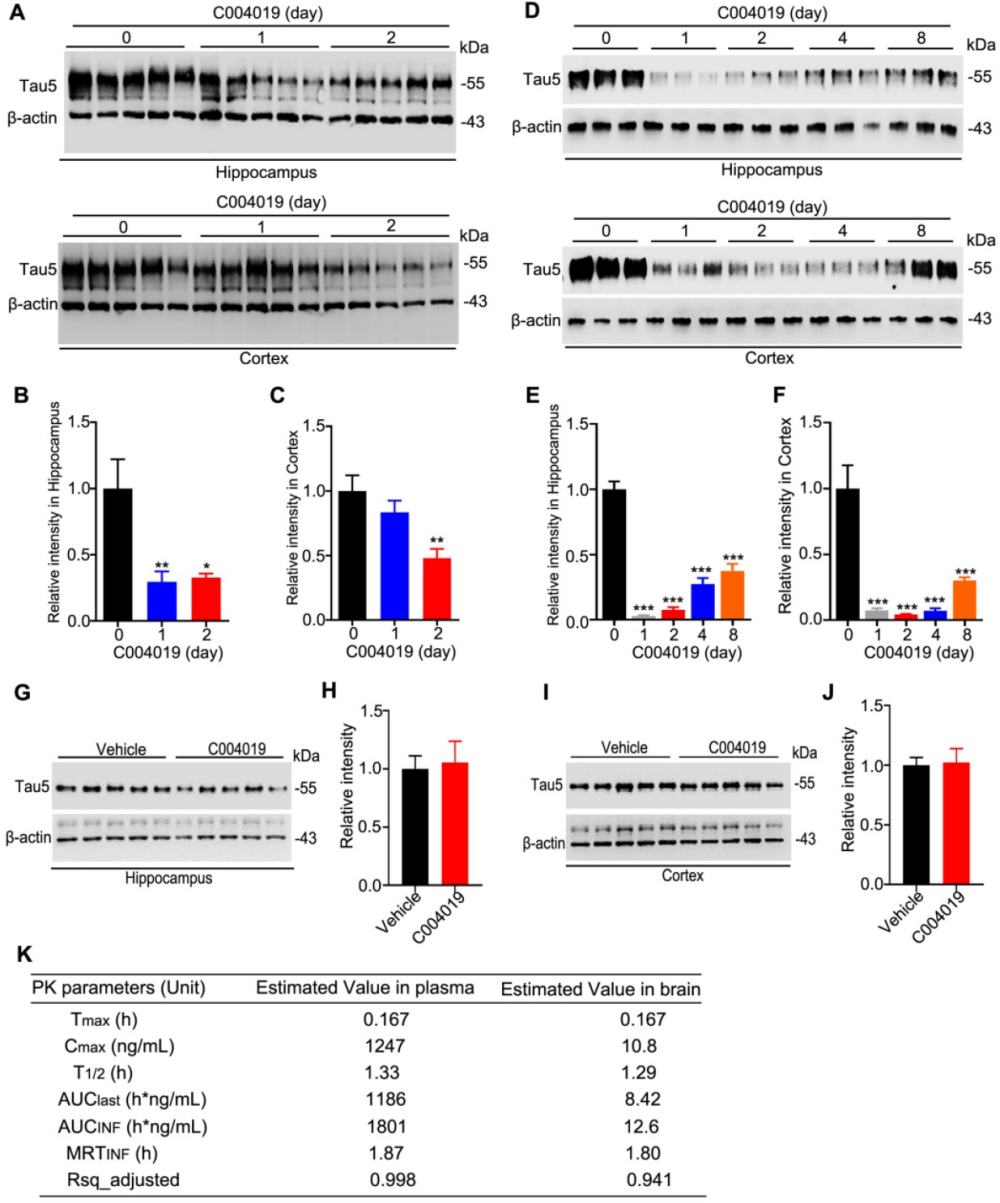
** Single-dose subcutaneous administration of C004019 induced a sustained tau reduction in the brain of wild-type mice.** C004019 was administrated in 4 m-old wild-type mice through subcutaneous injection, or oral gavage for different periods as indicated in the figure, and then the hippocampus or cortex extracts were prepared for Western blotting. **(A-C)** Subcutaneous injection of C004019 (15 mg/kg) induced tau reduction in the hippocampus and the cortex. **(D-F)** Single-dose subcutaneous injection of C004019 (3 mg/kg) induced a sustained tau reduction in the brain. **(G-J)** Oral gavage of C004019 (20 mg/kg) did not induce significant changes of tau proteins in hippocampus and the cortex measured at 48 h by Western blotting. **(K)** C004019 could penetrate to the brain of C57BL/6 mice after subcutaneous administration at a dose of 3 mg/kg measured by LC-MS/MS (N = 3 per timepoint). Tmax: the time point at which the drug concentration was the highest; Cmax: the maximum drug concentration; T_1/2_: half-life period of the compound; AUClast: area under the curve (the integral from the beginning to the last point in time); AUCinf: the integral from the beginning to infinity, i.e. the total area under the curve; MRTinf: the dose eliminated 63.2% of the time taken; Rsq_adjusted: the value of R^2^. Data were expressed as mean ± SEM (n = 5 for each group), (B, C, E, F) *P < 0.05, **P < 0.01, ***P < 0.001 vs. 0 day. Data were analyzed by one-way ANOVA. (H, J) Data were analyzed by Student's t-test.

As direct intracerebral drug delivery has limited application in the clinic, we tested whether periphery administration of C004019 could induce tau clearance in the brain of 4 m-old wild-type mice. C004019 (3 mg/kg, or 15 mg/kg) was injected into the mice through subcutaneous, and tau level in hippocampus and the cortex was detected by Western blotting at different time points. Significant tau reduction was detected in hippocampus and cortex at 24 h and 48 h after subcutaneous administration of C004019 at 15 mg/kg (Figure 3A-C). Unexpectedly, subcutaneous administration of low dose C004019 (3 mg/kg) for 24 h induced a much more significant tau reduction than that of high dose (15 mg/kg) in both hippocampus and the cortex (Figure 3D-F). Furthermore, single-dose administration of C004019 (3 mg/kg) induced a sustained tau reduction till 8 days, though a restoring tendency of tau level with time was shown (Figure 3D-F). We also tested the effect by intragastrical gavage (20 mg/kg, 48h) of C004019, but no significant tau reduction was detected (Figure 3G-J). To further identify if C004019 could penetrate the blood-brain barrier, we tested the time-dependent concentration of C004019 in plasma and brain after subcutaneous administration using liquid chromatograph-mass spectrometry/mass spectrometry (LC-MS/MS). The maximal brain concentration of C004019 was 10.8 ng/mL at 0.167 h and T_1/2_ was 1.29 h after subcutaneous administration at 3 mg/kg. The ratio of brain tissue maximal concentration of C004019 to the plasma maximal concentration (Kpuu value) was 0.00866 (Figure 3K). These data together demonstrated that subcutaneous administration of C004019 at 3 mg/kg can induce a robust and sustained tau clearance, though the brain concentration of C004019 was low. Therefore, subcutaneous injection of C004019 at 3 mg/kg was applied for the rest of the studies.

### Multiple-doses subcutaneous administration of C004019 promotes tau clearance with improved cognitive and synaptic functions in hTau and 3xTg-AD mice

**Figure 4:**
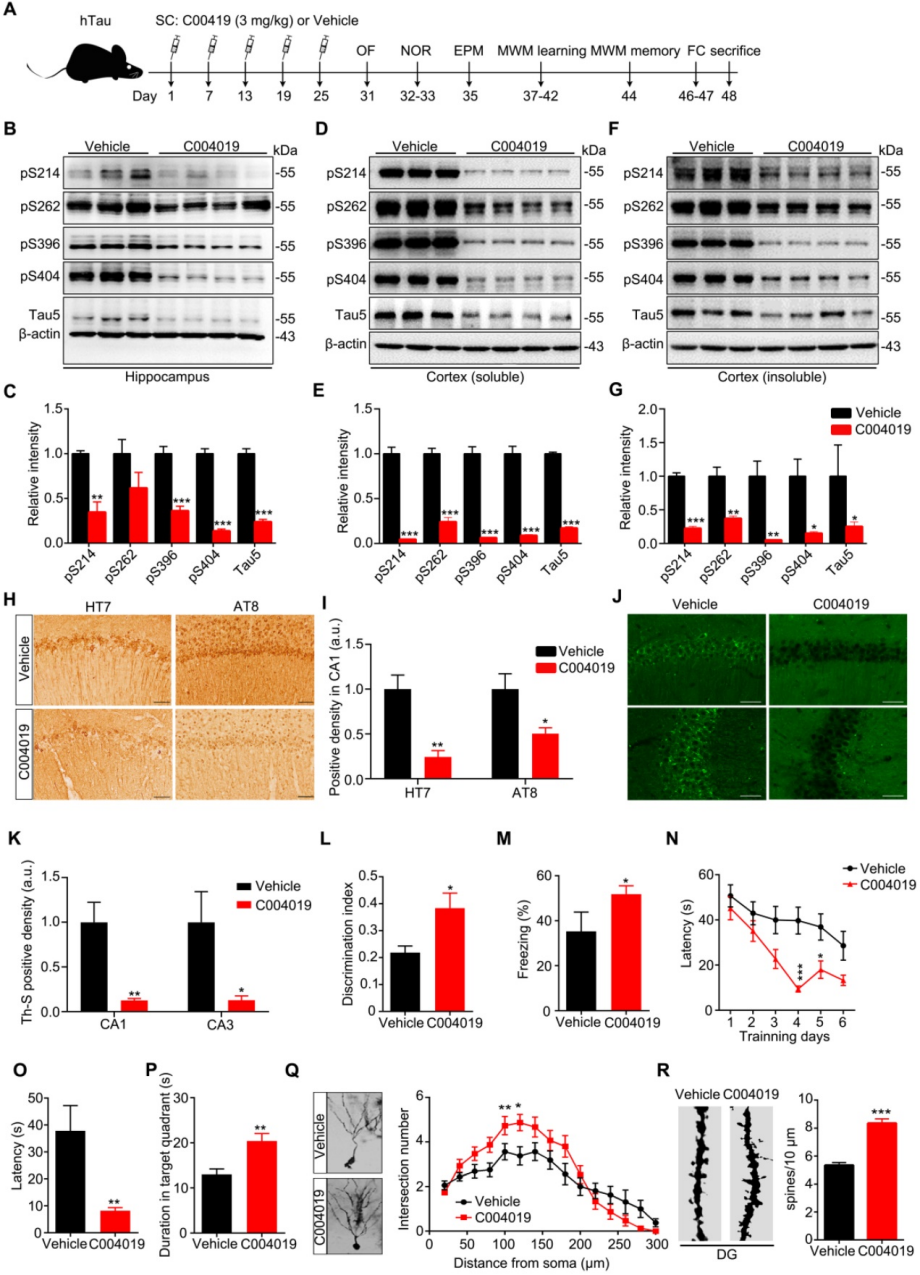
** Multiple-doses subcutaneous administration of C004019 induced tau clearance with improved cognitive function and dendritic morphology in hTau transgenic mice. (A)** Schematics showing treatment procedures, C004019 (3 mg/kg) or the vehicle was injected subcutaneously every 6 days for one month in hTau mice (12 m-old). After cognitive functions were measured, the hippocampus and cortex extracts or brain slices were prepared. **(B-G)** Remarkable reduction of total tau and p-tau in hippocampi and cortex (soluble and insuble fractions) of C004019-treated group was shown by Western blotting. **(H-I)** C004019 significantly decreased human tau (HT7), p-tau (AT8) measured by immunohistochemical (Scale bar: 50 µm). **(J-K)** C004019 significantly decreased aggregated tau measured by Thioflavin S staining (Scale bar: 50 µm). **(L)** C004019 treament improved cognitive functions. In NOR test, C004019 increased new object discrimination index. **(M)** In FC test, C004019 improved freezing index. **(N-P)** In MWM test, C004019 decreased latency to find the platform during learning trial (day 4 and day 5) and latency to reach the platform region and increased duration time in the target quadrant. **(Q-R)** C004019 increased neurite arborization (Q) and spine density (R) measured by Golgi-cox staining and Sholl analysis. For neurite arborization, at least 15 neurons from four mice per group were analyzed using two-way analysis of variance (ANOVA) with Bonferroni's multiple comparisons test. For spine density quantification, at least 50 neurons from hTau mice per group were analysed by Student's t-test. Data were expressed as mean ± SEM, (C, E, G, I, K, L, M, O, P, R)*P < 0.05, **P < 0.01, ***P < 0.001 vs. Vehicle. Data were analyzed by Student's t-test. (N) *P < 0.05, ***P < 0.001 vs. Vehicle. Data were analyzed by two-way ANOVA (n = 5 ∼ 6 for each group).

Known that single-dose subcutaneous administration of C004019 could reduce tau in normal brain, we further studied whether multiple-doses subcutaneous administration could be effective in the AD mouse models. In 12 m-old hTau transgenic mice, C004019 (3 mg/kg) or the vehicle was injected subcutaneously once every 6 days for a total of 5 times lasting for one month, then the cognitive functions were tested sequentially as indicated and the mice were sacrificed at day 48 after the first C004019 administration (Figure 4A).

Compared with vehicle control, the infrequent subcutaneous administration of C004019 significantly reduced total and p-tau levels in the hippocampal extracts (Figure 4B-C). This result further confirms that C004019 can induce a sustained reduction of tau. By fractionating the cortex extracts and Western blotting analyses, we observed that infrequent subcutaneous delivery of C004019 could reduce both soluble and insoluble tau, more efficiently to the tau in the soluble fraction (Figure 4D-G). Reduction of p-tau and tau aggregation in C004019-treated group were also shown by immunohistochemical staining using HT7 and AT8 (Figure 4H-I) and Thioflavin S staining (Figure 4J-K).

Previous studies demonstrated that 12 m-old hTau transgenic mice had memory deficits 21. We also observed that infrequent subcutaneous administration of C004019 attenuated remarkably the cognitive deficits in the mice, shown by the increased time exploring new object in novel object recognition (NOR) test (Figure 4L), and improved freezing index in fear condition (FC) test (Figure 4M), and the decreased latency to find the platform during six days learning trial or to reach the platform quadrant during probe trial in Morris water maze (MWM) test (Figure 4N-P). Meanwhile, no significant changes were detected in free movement (OF) (Figure S2A-C), duration time in the open arm (EPM) (Figure S2D-E), suggesting that the mice did not show changes in motor ability and anxiety level. By Golgi-cox staining and Sholl analysis, we observed that the neurite arborization and spine density in hippocampal DG significantly increased in C004019 group compared with the vehicle controls (Figure 4Q-R). These data together indicate that infrequent subcutaneous administration of C004019 can ameliorate cognitive functions and dentritic plasticity in hTau transgenic mouse model.

**Figure 5:**
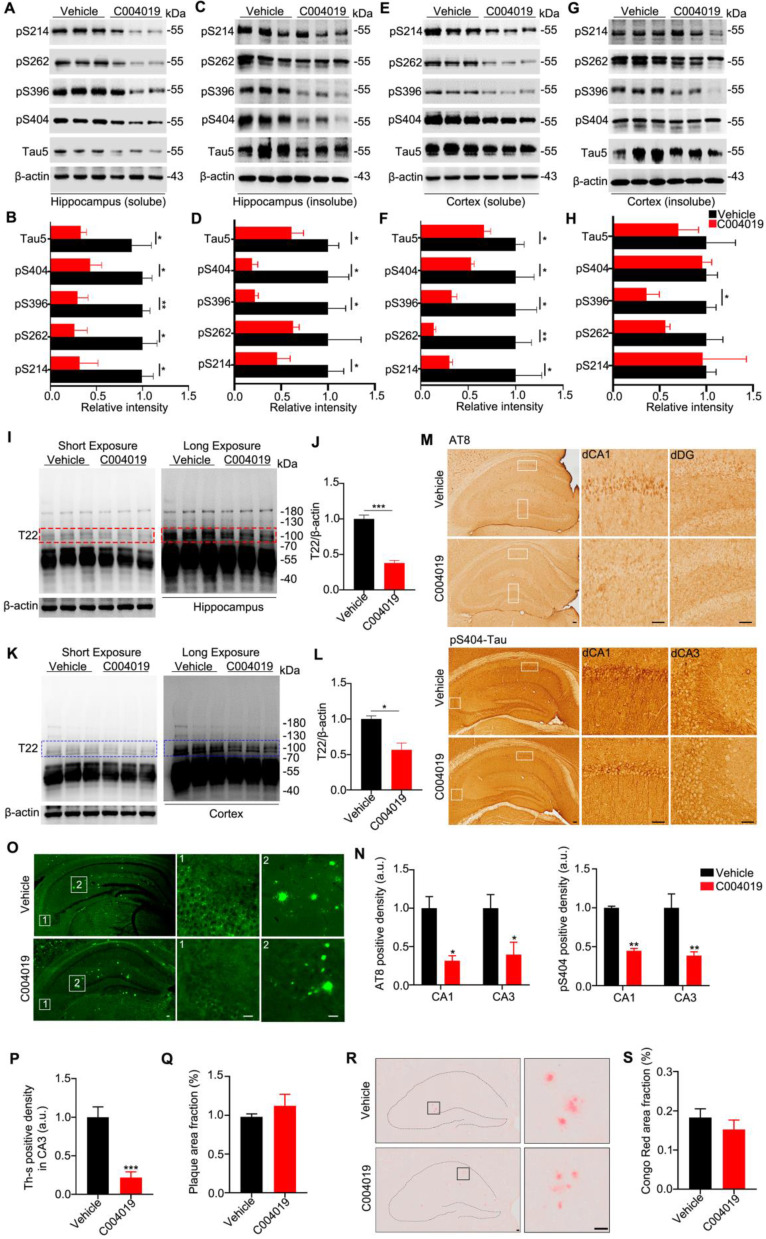
** Multiple-doses subcutaneous administration of C004019 induces tau clearance in 3xTg-AD mice**. C004019 (3 mg/kg) or the vehicle was injected subcutaneously into 9.5 m-old 3xTg AD mice (every 6 days for a total of 5 times in one month), and then different types of tau proteins were measured. **(A-H)** C004019 decreased both soluble and insoluble tau proteins in hippocampal and cortex extracts measured by Western blotting. **(I-L)** C004019 decreased oligomeric tau in hippocampus and cortex extracts measured measured by Western blotting using antibody T22. **(M-N)** C004019 decreased p-tau (AT8 and pS404) measured by immunohistochemical (Scale bar: 50 µm). **(O-Q)** C004019 decreased the aggregated tau levels measured by Thioflavin S staining (Scale bar: 50 µm) with no significant effects on plague. **(R-S)** C004019 had no significant effects on Aβ plaque measured by Congo red staining (Scale bar: 50 µm). Data were expressed as mean ± SEM, *P < 0.05, **P < 0.01, ***P < 0.001, vs. Vehicle. Data were analyzed by Student's t-test.

Using the same procedure, we further studied the effects of C004019 on 9.5 m-old 3xTg-AD mice, which harbor both tau and Aβ pathologies 19, 20. C004019 remarkably reduced both soluble and insoluble tau levels in hippocampal and cortex extracts, more efficiently to the tau in the soluble fraction (Figure 5A-H). By using T22, an antibody reacts with oligomer tau 22, we found that C004019 significantly reduced T22-positive tau in the soluble fraction (Figure 5I-L). It should be noted that the antibody T22 used in the present study could detect both low (~ 55 KDa) and high molecular weight (100 ~ 250 KDa) tau proteins; as the band at ~ 110 KDa was not dark enough (Figure 5I, K, left panel), we enhanced the band by longer exposure (Figure 5I, K, right panel) and we specualted that these tau species might be the dimeric tau. Reduction of p-tau and aggregated tau proteins in C004019-treated group was also shown by immunohistochemical staining using pS404 and AT8 (Figure 5M-N) and Thioflavin S staining (Figure 5O-Q). No significant effects on amyloid-β plaque was detected by Congo red staining (Figure 5R-S). These data further confirm that infrequent subcutaneous administration of C004019 could promote clearance of tau proteins in AD mice.

**Figure 6:**
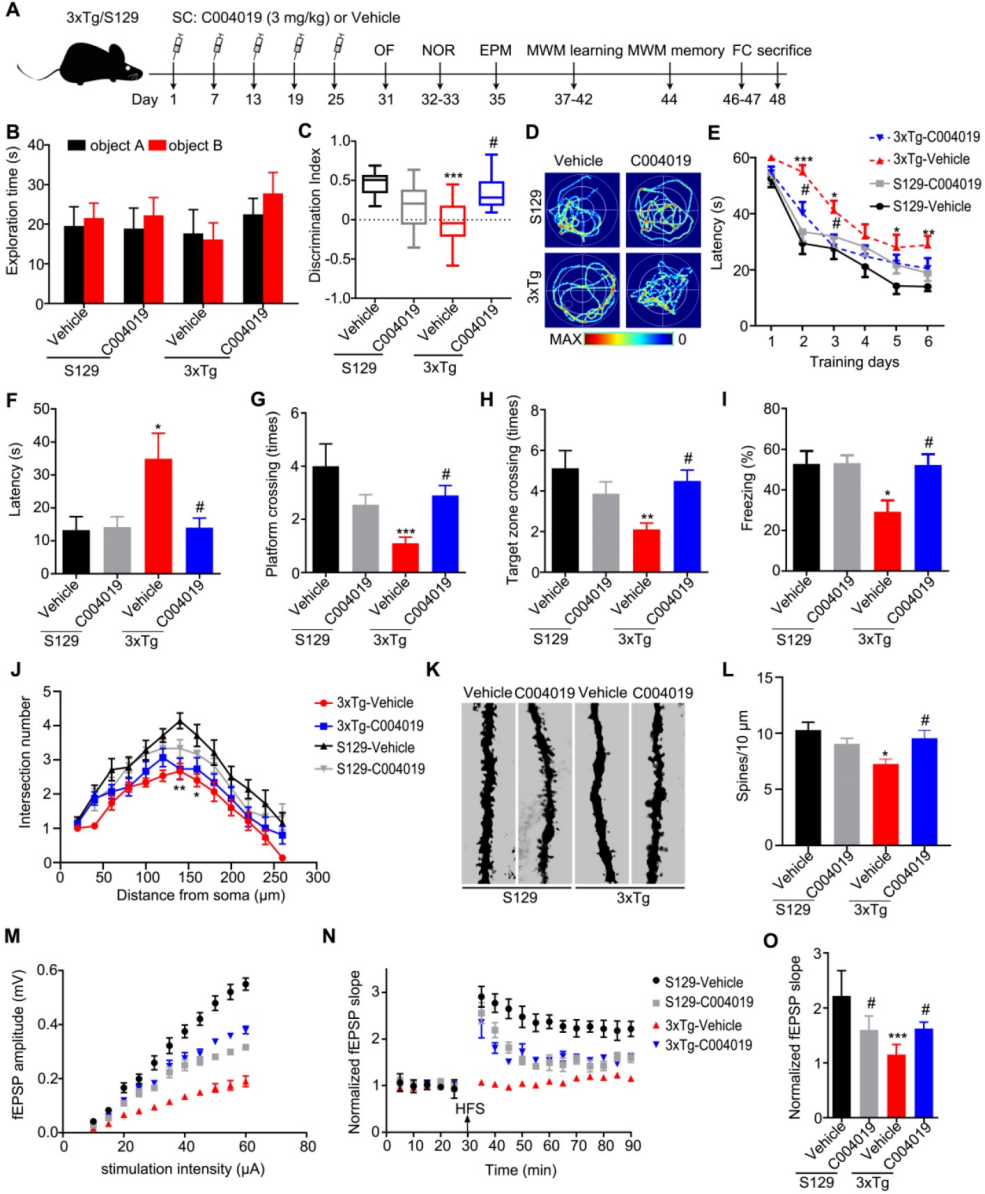
** Multiple-doses subcutaneous administration of C004019 attenuated the cognitive and synaptic deficits in 3xTg-AD mice. (A)** Treatment procedure: C004019 (3 mg/kg) or the vehicle was injected subcutaneously every 6 days for one month in 9.5 m-old 3xTg-AD or S129 mice, and then the cognitive behaviors were detected. **(B-C)** C004019 treatment increased the discrimination index in 3xTg mice measured by NOR. **(D-H)** In MWM test, C004019 decreased the latency to find platform during learning trial and increased the latency, platform crossings and target zone crossings during the probe trial. **(I)** C004019 increased the freezing index in 3xTg mice during FCT. **(J)** C004019 didn't affect neurite arborization measured by Sholl analysis. **(K-L)** C004019 increased spine density measured by Golgi-cox staining (at least 20 neurons from 3 ∼ 4 mice for each group were analysed for spine density). **(M-O)** C004019 rescued synaptic functions as shown by an increased input-output response with potentiation of LTP indicated by an increased slope of the evoked fEPSP; the increase was still significant at 60 min after high frequency stimulation (HFS) (n = 8 hippocampal slices from 3 ~ 4 mice in each group). Data were expressed as mean ± SEM (n = 9 ∼ 10 for each group), *P < 0.05, **P < 0.01, ***P < 0.001 vs. S129-Vehicle. #P < 0.05 vs. 3xTg-Vehicle. Data in (B, C, F-I, L, O) were analyzed by one-way ANOVA. Data in (E, J) was analyzed by two-way repeated-measures ANOVA.

Although the vehicle-injected 3xTg mice showed shorter movement distance and longer center duration than the other three groups of mice during OF test, the changes had no statistical significance (Figure S3A-B). Additionally, the four group of mice did not show difference in EPM test (Figure S3C). These data suggest that 3xTg mice had neither motor dysfunction nor anxiety behavior, and C004019 treatment did not significantly affect the motor and anxiety levels of the mice. The 3xTg mice showed learning and memory deficits when compared with age-matched S129 control mice, while C004019 treatment rescued the cognitive functions measured by NOR, MWM and FC tests (Figure 6B-I). Specifically, the 3xTg mice showed decreased discrimination index during NOR test compared with the age-matched S129 controls, while C004019 treatment almost completely restored the memory ability (Figure 6B-C). In MWM test, the 3xTg mice treated with C004019 showed shorter latency to reach the platform during 6 days learning trial, and an decreased latency to reach the platform, increased platform crossings and target zone crossings during the probe trial compared with the vehicle-treated 3xTg mice (Figure 6D-H), indicating a rescue of spatial learning and memory by C004019. In FC test, C004019 treatment restored the freezing index in 3xTg mice (Figure 6I). Additionally, the 3xTg-AD mice showed reduced spine numbers and impaired LTP induction, while infrequent subcutaneous administration of C004019 restored spine density (Figure 6K-L) and LTP induction, including fEPSP amplitude and slop (Figure 6M-O), but didn't have any effect on neurite arborization (Figure 6J). These data together confirm that periphery administration of C004019 is indeed effective in improving the cognitive and synaptic functions in 3xTg-AD mice.

## Discussion

Increasing evidence suggests that intracellular accumulation of tau plays a pivotal role in neurodegeneration and memory deficits in AD and the related neurodegenerative disorders, collectively termed tauopathies 23, 24. Therefore, developing drugs aimed at tau clearance have caught tremendous attention recently.

Targeted protein degradation using PROTAC to degrade specific proteins from within cells is a novel drug discovery strategy 14, 25. As PROTACs could potentially be generated for any protein including a large subset of 'undruggable' proteins, it may be used to target tau, a natively unfolded undruggable protein. Indeed, several peptidic tau PROTACs have been reported 13, 15, 16. However, due to the intrinsic weakness of peptides including vulnerability to protease degradation and poor membrane permeability, the application of peptidic PROTAC* in vivo* is limited. Recently, several small-molecule tau PROTACs using a tau PET tracer as a warhead have been developed 16, but their application *in vivo* has not been reported.

Here, we designed a small-molecule PROTAC, named as C004019 with a molecular mass of 1,035.29 dalton. The chimera is composed of three parts including a tau binder, a linker and an E3 ligase recruiter or Vhl binder, in which the two functional parts respectively binds to tau proteins and E3 ligase to reach a selective and efficient ubiquitination and the subsequent proteasome-mediated proteolysis of tau. By *in vitro* and *in vivo* testing, we demonstrated that C004019 could efficiently induce clearance of tau proteins in both physiological and pathological conditions. Remarkably, even single-dose or infrequently (once per 6 days) subcutaneous administration of this molecule achieved a sustained tau reduction in the brain of hTau and 3xTg-AD transgenic mouse models. Lowing tau can alleviate Aβ-induced neurotoxicity 26, 27, and knockdown of tau does not elicit obvious abnormalities in mice 28, 29. Therefore, C004019 could be a promosing drug candidate for tauopathies, including AD.

It is well recognized that the proteasome's barrel-like structure does not allow large molecules, such as aggregated tau proteins, to enter directly proteasome was not capable of degrading protein aggregates 30. Thus, we particularly designed the chimera against total tau at apparent molecular weight ~ 55 KDa as warhead. Our results showed that although C004019 could efficiently reduce tau proteins in both soluble and insoluble fractions including the oligomeric tau, it was most effective to the tau in the soluble fraction which was consistent with the expected function of the tau binder. C004019 could induce the formation of a stable ternary complex, i.e., PROTAC, tau and E3 ligase. Importantly, C004019 significantly promoted poly-ubiquitination of tau, leading to proteasome-dependent clearance of tau. These results clearly show that C004019 truly exerts its function as an active PROTAC. Our data are also consistent with the other reports showing that a warhead with high affinity to target protein is not a prerequisite for generation of an active PROTAC, instead, inducing the formation of a steric structure by the target protein and hijacked E3 ligase allowing the transfer of ubiquitin from E2 to target protein is critical for a PROTAC to function properly 14.

Given that tau is a natively unfolded protein, it could be challenging to develop a small-molecule compound showing selectivity between different tau species. We noticed that C004019 reduced mouse and human tau to a similar extent without obvious selectivity in wild-type, hTau and 3xTg-AD mice. In addition, C004019 did not exhibit preferential degradation of the phosphorylated tau, as it decreased most of the phosphorylated tau to a similar extent as total tau, although there were variations of reduction between tau phosphorylated at different sites. Notably, C004019 induced a more pronounced reduction of soluble tau than insoluble tau in both hTau and 3xTg AD mice. As tau aggregates cannot be directly degraded by proteasome 31, 32, it is conceivable that their reduction could be a result of a slow shift of the equilibrium between aggregates and soluble monomers/oligomers toward the generation of monomers due to their clearance by C004019.

Interestingly, despite of the comparably less reduction of insoluble tau, C004019 nearly completely rescued the behavioral deficits in both hTau and 3xTg-AD mice. These results were consistent with earlier reports showing that it is the soluble tau species instead of tau aggregates that cause neurodegeneration 33, 34. Synapse loss is well correlated with severity of dementia in AD, and tau hyperphosphorylation plays a key role in impairment of synaptic plasticity 35, 36. We found that C004019 significantly alleviated the dendrite loss in hTau and 3xTg AD mice, which might be due to the dramatic reduction of hyperphosphorylated tau. The improvement of behavioral performance in hTau and 3xTg-AD mice upon C004019 treatment is likely an outcome of the recovery of synaptic plasticity.

Due to the comparably larger size than most brain-active drugs, PROTACs likely suffer from poor blood-brain barrier permeability. However, to our surprise, subcutaneous administration of C004019 could induce a robust tau clearance in the brains of mice. C004019 is a potent tau degrader with a DC50 of ~5 nM in cultured cells, therefore, it could induce significant tau clearance even a very low level of C004019 entered the brain. Of note, we observed that a dose of 15 mg/kg showed less efficacy than a dose of 3 mg/kg in mice. Whether this is due to the “hook effects” is not clear. Up to date, the level of Vhl in neurons remains unknown. Since tau is a protein of high abundance in neurons with an estimated concentration of 1-2 μM, it seems unlikely that the amount of C004019 can achieve a level much higher than tau to elicit hook effects. However, given that over 99% of tau is bound to the microtubule in physiological condition, the free tau in neurons may be actually pretty low. Thus, the possibility of the occurrence of hook effects induced by C004019 cannot be fully excluded. Further experiments are needed to address this issue.

One advantage of PROTAC over traditional small-molecule inhibitors is that its action model is event-driven, which is distinctive from the occupancy-driven mechanism of the traditional small-molecule inhibitors. This could offer PROTACs an advantage in cases where the synthesis of target protein is slow. In such cases, PROTACs can provide sustained *in vivo* efficacy even long after the PROTACs have been cleared, which enables an infrequent dosing regimen. Tau is a long-living protein 37. We found that tau was still reduced 50% at 8 days after one treatment with C004019 in wild-type mice. Furthermore, infrequent dosing (once per 6 days) of C004019 for one month achieved good efficacy. It has been reported that the half-life of human brain tau is about 23 days 37. This indicates that if used for treatment in patients, the potential dosing frequency of C004019 might be once per 2-3 weeks or even longer. Although oral administration appears to be the ideal drug delivery route, an infrequent subcutaneous dosing e.g. once per 2-3 weeks would be acceptable for patients. In fact, due to the comparable larger size of PROTACs, it is a big challenge to develop a PROTAC with oral bioactivity. Thus, C004019 might not necessarily undergo further optimization to achieve oral bioactivity.

Taken together, we successfully developed a small-molecule tau PROTAC named as C004019, which can selectively and efficiently induce tau clearance with remarkably improved synaptic and cognitive functions in different hTau-cell models, and in AD-like hTau and 3xTg transgenic mice. Therefore, C004019 represents a promising drug candidate for AD and the related tauopathies.

## Materials and methods

### Antibodies, plasmids and chemicals

Antibodies used in the present study include polyclonal antibodies (pAbs) anti-pS214 (Signalway Antibody, 11109), anti-pS262 (Signalway Antibody, 11111), anti-pS396 (Signalway Antibody, 11102), anti-pS404 (Signalway Antibody, 11112), T22 (anti-Tau, Millipore, ABN454), anti-GFP (Santa Cruz Biotechnology, FO115), and anti-Microtubule-Associated Protein 2 (MAP2) (Millipore, AB5622). Moloclonal antibodies (mAbs) Tau5 (MAPT/Tau, Abcam, ab80579), anti-Ub (P4D1) (Santa Cruz Biotechnology, C3017), anti-Vhl (Cell Signaling Technology, 68547), HT7 (ThermoFisher Scientific, MN1000), AT8 (Thermo Fisher Scientific, MN1020), and anti-β-actin (Abclonal, AC026). Plasmids including pEGFP-hTau-2N4R and 3xFlag-hTau were sequenced and prepared using an endotoxin-free plasmid extraction kit (Tiangen). MG132 (MedChemExpress, HY-13259, Molecular Weight: 475.62, 10 mM) was dissolved in 99.7% dimethyl sulfoxide (DMSO, Sigma-Aldrich, D2650). Hydroxypropyl-β-cyclodextrin (HP-β-CD, Sigma-Aldrich, 778966, 20%) used as co-solvent and antidote for C004019 was dissolved in normal saline. The C004019 powder and Tau binder powder were synthesized and provided by Neurosmart Therapeutics Co., Ltd. The C004019 stock aliquots (50 mM dissolved in 99.7% DMSO, 10 μL, molecular weight: 1,035.29) were stored at -80 ºC, and diluted with 20% HP-β-CD to the indicated final concentrations before use. Tau binder stock aliquots (50 mM dissolved in 99.7% DMSO, 10 μL, molecular weight: 375.5) were stored at -80 ºC. GF-109203X (GFX, MedChemExpress, HY-13867, Molecular Weight: 412.48) stock aliquots (10 mM dissolved in 99.7% DMSO, 10 μL) were stored at -80 ºC. Wortmannin (WO, MedChemExpress, HY-10197, Molecular Weight: 428.43) stock aliquots (10 mM dissolved in 99.7% DMSO, 10 μL) were stored at -80 ºC. WO alone could activate GSK-3β, and combination of WO with GFX significantly enhanced the effect of GSK-3β activation by simultaneous inhibiting phosphoinositol-3 kinase and protein kinase C, the upstream kinases that can phosphorylate GSK-3β at Serine-9 and thus inhibits the activity of the kinase 17, 18, 38, 39.

### Cell culture, transfection and treatments

SH-SY5Y human neuroblastoma cells were cultured in 90% DME/F-12 medium (HyClone, SH30023.01) with 10% fetal bovine serum (FBS) (Biological Industries, 04-001-1ACS) and penicillin (100 U/mL)/ streptomycin (100 μg/mL) in a 37 °C incubator with humidified atmosphere of 5% CO2. Human embryonic kidney 293 cells (HEK293) or HEK293 with stable expression of wild-type full-length human tau (termed as HEK293-hTau) were cultured in 90% DMEM/High Glucose medium (HyClone, SH30022.01) containing 10% FBS and 200 μg/mL G418 (ThermoFisher Scientific, 10131027), in a humidified atmosphere of 5% CO2 at 37 °C. Neofect (DNA transfection reagent, TF20121201) was used for plasmids transfection. The cells were placed onto 6-well or 12-well plates. Plasmids (EGFP-hTau or 3xFlag-hTau or the vector), neofect and Opti-MEM medium (Gibco, 31985-070) were made into a mixture in a ratio of 1 μg: 1 μL : 100 μL and then added into each well of the plate. To inhibit proteasome activity, HEK293-hTau cells were cultured with DMEM/High medium containing 20 μM C004019 with or without 10 μM MG132, or the same volume of vehicle (DMSO) for 24 h at 37 °C. To induce tau hyperphosphorylation, SH-SY5Y cells were cultured with DME/F-12 medium containing 10 μM C004019 with or without 1 μM GFX and 1 μM WO for 24 h at 37 °C.

### Cell viability analysis

Cell viability was assessed using CCK-8 kit by following the manufacturer's instructions. The cells were seeded at a concentration of 5,000 cells per well in a 96-well plate and then treated with various concentrations of C004019 (0, 0.01, 0.1, 1, 10, 20 and 100 μM) for 24 h. After the treatments, the culture medium was removed, and 10 μL of CCK-8 in 90 μL of medium was added. After incubating for 30 min at 37 °C, the absorbance was measured at 450 nm using a microplate reader (BioTek, 250058).

### Animals and drug administration

Male C57BL/6 mice (2 m-old, 20 ± 5 g) were purchased from the Experimental Animal Central (Beijing Vital River Laboratory Animal Technology Co., Ltd.). The human tau (hTau) transgenic mice (*Mapt^tm1(EGFP)Klt^*Tg(MAPT)8cPdav/J, stock number 004808) and 3xTg-AD mice harboring knock-in of the Swedish double mutation of amyloid precursor protein (APP), a presenelin 1 mutation (*Psen1^tm1Mpm^*), and a frontotemporal dementia tau mutation (tauP301L) (B6;129-Tg(APPSwe,tauP301L)1Lfa *Psen1^tm1Mpm^*/Mmjax, stock number 34830) were kind gifts of Dr. Xifei Yang (Laboratory of Modern Toxicology of Shenzhen, Shenzhen Center for Disease Control and Prevention, Nanshan District, Shenzhen, China). All mice were kept at 24 ± 2 ºC with accessible food and water under a 12 h light/dark cycle. All animal experiments were approved by the Ethics Committee of Tongji Medical College.

For brain surgery and stereotaxic cerebroventricular drug delivery, the mice were placed in a stereotaxic apparatus and anesthetized with 2% isoflurane (RWD Life Science, R510-22) through a nose cone (300-500 mL/min, RWD Life Science, China, R500). The scalp was incised along the midline between the ears after the mice were sterilized with iodophors and 75% (vol/vol) alcohol. The holes were drilled stereotaxically in the skull at a cerebroventricular location (posterior 0.22 mm, lateral 0.9 mm, and ventral 2.3 mm relative to bregma). Using a microinjection system (Shenzhen RWD Life Technology Co., Ltd; RWD 69100), C004019 (5 μL, 200 μM) was infused at a rate of 0.5 μL/min. The needle was kept in place for 10 min before withdrawal. After the skin was sutured, the mice were placed on a heater for analepsia. For subcutaneous injection, C004019 was diluted with 20% HP-β-CD and injected under the skin on the back neck to reach a final concentration of 3 mg/kg (low dose) or 15 mg/kg (high dose).

### Western blotting

The cells or hippocampal/cortex tissues were homogenized for ~ 30 min on ice in buffer [50 mM Tris·HCl pH 7.4-7.5, 100 mM NaCl, 1% (vol/vol) Triton X-100, 5 mM EDTA, 1:100 PMSF, 1:1,000 protease inhibitor cocktail containing 4-(2-Aminoethyl)-benzenesulfonyl fluoride hydrochloride, aprotinin, bestatin, leupeptin, E-64, and pepstatin A stored at 4 °C], and then centrifuged at 12,000 × g at 4 °C for 20 min. The supernatant was collected and the protein levels were analyzed using bicinchoninic acid (BCA, KF016, Sigma-Aldrich) by following the manufacturer's instruction (served as soluble protein). The pellet was further incubated with 8% (wt/vol) SDS buffer at 4 °C and then treated by ultrasonic wave to reach a complete resuspension. The suspension was spun (18,000 × g, 4 °C for 20 min) and the supernatant collected (served as insoluble fraction). The supernatant of different faction was respectively mixed with loading buffer (3:1, vol/vol) containing 200 mM Tris-HCl, pH 6.8, 8% SDS, 40% glycerol, and boiled for 10 min. For Western blotting, the proteins were separated by 10% SDS-polyacrylamide gel via electrophoresis for about 2 h and transferred to nitrocellulose membranes (0.45 nm) for 1 h. The membranes were blocked with 5% (wt/vol) BSA dissolved in PBS for 1 h and incubated with primary antibodies overnight at 4 °C in cold room. Membranes were then incubated with a secondary antibody for 1 h at room temperature and visualized using the ECL Imaging System (610007-8Q, Clinx Science Instruments Co., Ltd). Immunoreactive bands were quantitatively analyzed by Image J2x software.

### Immunohistochemistry

For immunohistochemical studies, mice were anesthetized and then perfused through the aorta with 100 mL 0.9% normal saline followed by 400 mL phosphate buffer containing 4% paraformaldehyde prepared one day before. Brains were removed and postfixed in perfusate overnight and then immersed into 30% (wt/vol) sucrose liquid diluted with PB twice for about 3 days. Once sinking to the bottom of the tubes, brains were snap frozen at -80 °C and then cut for sections (30 μm) with a freezing microtome. The sections were collected consecutively in PBS or antifreezing solution (PBS: glycerin: ethylene glycol = 5:2:3, vol/vol/vol) for immunohistochemistry staining. Free floating sections were blocked with 0.3% H2O2 diluted in PBS in dark for 30 min and nonspecific sites were blocked with 5% BSA for 30 min at room temperature. Then sections were incubated with primary antibodies overnight at 4 °C. After washing with PBS three times, sections were subsequently incubated with biotin-labeled secondary antibodies for 1 h at 37 °C. Next, sections were pasted onto overhand slides previously coated with gelatin, staying overnight to dry completely. At last, they were sequentially dehydrated in 50%, 75%, 95% and 100% ethanol for 6 times (20 min each) and cleared in xylene for 3 times (30 min each) and cover-slipped with Permount solution. The immunoreaction was developed using the Polink-2 plus® Polymer HRP Detection kit (ZSGB-BIO, PV-9001/PV9002) according to manufature's instruction and observed under a microscope. For each primary antibody 3 to 5 consecutive sections from each brain were used.

### Immunofluorescence

Brain sections and cultured cells were fixed in 4% (vol/vol) paraformaldehyde for 30 min at room temperature and permeabilized in 0.5% Triton X-100 (vol/vol) diluted in PBS solution. Nonspecific binding sites were blocked via incubating in 5% (wt/vol) BSA containing 0.1% Triton X-100 (vol/vol) for 30 min. The samples were incubated with primary antibodies at 4 °C overnight, followed by washing 3 times in PBS and subsequent incubation with secondary antibodies conjugated to Alexa-Fluor 488 for 1 h at 37 °C. After washing 3 times in PBS, the samples were further stained with hoechest (1:1,000) for 10 min. Finally, samples were washed and mounted onto slides with 50% glycerin-PBS (vol/vol) solution. All the slides were imaged with a confocal microscope (Zeiss Carl LSM 780, Germany).

### Immunoprecipitation

Cell lysates were centrifuged at 12,000 g for 20 min at 4 °C and the supernatant were incubated with protein G agarose beads (Millipore, 16-266), pre-washed with cold PBS for 1 h at 4 °C to remove non-specific proteins. After centrifuged (14,000 g) at 4 °C for 15 min, the supernatant was collected and protein concentration was measured by BCA assay. The supernatant was sequentially incubated with primary antibodies (2 μg/100 μg) overnight at 4 °C with rotating in the first day and protein G agarose beads (20 μL/100 μL) the next day. After spin (6,000 g) for 2 min at 4 °C, the pellets were collected, washed with precooled washing buffer for 3 times, resuspended in buffer (50 mM Tris-HCl, pH 6.8, 2% SDS, 10% glycerol), boiled for 10 min, and analyzed by Western blotting.

### Quantitative real-time PCR

Total RNA was isolated by using Trizol™ kit (Invitrogen, Carlsbad, CA, USA) and the reverse transcription reagent kit (RR037, Takara) was used to obtain cDNA. RT-PCR was performed using a StepOnePlus Real-Time PCR Detection System (AB Applied Biosystems, 272001262, Cossell Biotechnology). The PCR system contains 2 μL forward and reverse primers, 10 μL SYBR Green PCR master mixes, 1 μL cDNA and 7 μL diethylpyrocarbonate (DEPC H2O). The primer of MAPT-forward was CAGCTCCGGCACCAACAG and the MAPT-reverse was CCTGGTTCAAAGTTCACCTGAT. The primer of β-actin-forward was GAGACCTTCAACACCCCAGC and β-actin-reverse was GGAGAGCATAGCCCTCGTAGAT.

### Open field test

The Open field (OF) test was consisted of a 5 min session in a quadrate chamber (white opaque plastic). The chamber was divided into 16 square regions, a central field (center 4 square regions) and a periphery field. Each mouse was placed in the same position at the start of the test. Behaviors were recorded and analyzed by the video tracking system (Chengdu Taimeng Software Co., Ltd, China).

### Novel object recognition

For novel object recognition (NOR) test, the mice were habituated to a quadrate chamber (white opaque plastic) for 5 min without objects 24 h prior to the test. The chamber was cleaned with 75% ethanol between each habituation period. The day after the mice re-entered the chamber from the same starting point and were given 10 min to familiarize themselves with the object A and object B. After each period the chamber and objects were cleaned with 75% ethanol. The next day after the familiarization period, object B was replaced with noval object C, and the mice were given 10 min to explore both objects. The exploring time in object A (TA) and C (TC) was recorded using the video tracking system. The discrimination index was calculated by (TC-TA)/(TA+TC).

### Elevated plus maze

The Elevated plus maze (EPM) test was performed as follows: mice were placed at the junction of the four arms of the maze (white opaque plastic), and started the test from facing an open arm. The chambers were cleaned with 75% ethanol before the next mouse was trained. The entries and duration time in each arm were recorded for 5 min by the video tracking system (Chengdu Taimeng Software Co., Ltd, China). An increase in open arm activity (duration and/or entries) reflects anti-anxiety behavior.

### Morris water maze

The Morris water maze (MWM) test was conducted to assess the spatial learning and memory. The maze was filled with water and divided into 4 quadrants with a platform placed in one quadrant. The mice were trained to find the hidden platform for 6 consecutive days, and underwent 3 training trials (once per quadrant) per day from 14:00 to 20:00 pm. In each training trial, the mouse started from one of four quadrants facing the wall of the pool and the trial ended when the animal climbed on the platform. If mice failed to locate the platform within 60 s, they were gently guided onto the platform and stayed there for 30 s; the escape latency was recorded as 60 s. The spatial memory was tested 1 d after the last training by removed the platform. The longer a mouse stayed in the previous platform-located quadrant, the better it scored the spatial memory. A video camera, fixed to the ceiling 1.5 m from the water surface, was used to record the swimming path and the time for mice to find the platform (latency) or to pass through the previous platform-located quadrant each day. The camera was connected to a digital-tracking device attached to an IBM computer (Armonk, NY).

### Fear conditioning

For fear conditioning (FC) test, the mice were placed into a square chamber with a grid floor. On the first day (day 1), each mouse was habituated to the chamber for 3 min, and then a foot shock (0.9 mA, 3 s) was delivered. Three sequential foot shocks at 3 min intervals were applied. Then the mice were returned to their home cages. On the next day (day 2), the mice were exposed to the same chamber without any stimulus for 3 min. The contextual conditioning was assessed by recording freezing behavior during the 3 min exposure. Freezing time during the 3 min was recorded for assessment of memory.

### Electrophysiological recordings

Mice used for electrophysiology experiments were deeply anesthetized as described above. When all pedal reflexes were abolished, brains were removed and placed in ice-cold oxygenated slicing solution containing the following: 225 mM sucrose, 3 mM KCl, 1.25 mM NaH2PO4, 24 mM NaHCO3, 6 mM MgSO4, 0.5 mM CaCl2, and 10 mM D-glucose. Coronal slices (300 μm thickness) were cut at 4-5 °C in the slicing solution using a Leica VT1000S vibratome and then transferred to an incubation chamber filled with oxygenated slicing solution in a 30 °C water bath for 1 h before being recorded. For LTP, slices were laid down in a chamber with an 8 × 8 microelectrode array in the bottom planar (each 50 × 50 μm in size, with an interpolar distance of 150 μm) and kept submerged in artificial cerebrospinal fluid (aCSF; 1-2 mL/min) with a platinum ring glued by a nylon silk. Signals were acquired using the MED64 System (Alpha MED Sciences, Panasonic). The fEPSPs were recorded by stimulating the Schaeffer fibers. LTP was induced by applying three trains of high-frequency stimulation (HFS; 100 Hz, 1 s duration). LTP magnitude was calculated as the average (normalized to baseline) of the responses recorded 50-60 min after conditioning stimulation. All the signals were recorded by using the MED64 System.

### Golgi staining and spine analyses

Golgi staining was performed by using a Rapid GolgiStain Kit (FD neurotechnologies, PK401) according to the manufacturer's instructions. Briefly, the animals were sacrificed and perfused for 5 min with PBS. All procedures were performed under dark condition. Brains were dissected out and immersed in impregnation solution (equal volumes of Solutions A and B, containing mercuric chloride, potassium dichromate, and potassium chromate, mixed for 24 h in advance), and stored at room temperature. Impregnation solution was replaced after 24 h. After 4 weeks, brains were transferred to Solution C and stored at 4 °C for 72 h, with the solution replaced after 24 h. The brain was sectioned coronally (100 μm) using a vibrate microtome (VT 1000 s, Leica, Nussloch, Germany) and sections were mounted on gelatin-coated microscope slides with Solution C. Slides were rinsed twice in distilled water (2 min each), and then placed in a mixture of Solution D:E:distilled water (1:1:2, vol/vol/vol) for 10 min. After rinsing with distilled water, sections were dehydrated in 50%, 75%, 95% and 100% ethanol for 4 times (4 min each). Sections were cleared in xylene 3 times (4 min each) and coverslipped with Permount solution. The spine morphology was analyzed by Nikon microscope. The spine numbers per 10 μm of dendrite per neuron were calculated by using Image-Pro Plus 6.0, and 5 to 7 neurons per mouse (3 mice per group) were used for statistical analyses.

### Pharmacokinetics (PK) study

Male C57BL/6 mice (age of 6-8 weeks with bodyweight 21-23 g; N = 24) were purchased from JH Laboratory Animal Co. LTD. Subcutaneous injection dosing solution was prepared by diluting C004019 stock solution in 20% HP-β-CD in saline. The animals were administrated with C004019 at a dose of 3 mg/kg (10 mL/kg) via subcutaneous injection (N = 3 per timepoint), and then were anaesthetized via isoflurane at the designated time points, followed by collection of approximately 110 µL of blood sample into EDTA-2K tubes via retro-orbital puncture or cardiac puncture for terminal bleeding. The brain samples were collected at the designated time points after blood collection. All the samples were first maintained in wet ice and then the blood samples were centrifuged (2000 g, 4 °C, 5 min) to obtain plasma within 15 minutes post sampling. Afterwards, all the plasma samples and brain samples were stored at approximately -70 °C until analysis. The concentration of C004019 in plasma and brain was determined using liquid chromatograph mass spectrometry/mass spectrometry (LC-MS/MS) with a UPLC/MS-MS-015 (QTRAP 5500).

### Statistical analysis

All data were collected and analyzed in a blinded manner. Data were analyzed using Graphpad software. Statistical analyses were performed using Student's t test for two-group comparisons or one-way or two-way ANOVA, followed by post hoc tests for multiple comparisons among more than two groups. The results were presented as mean ± SEM and *P* < 0.05 was accepted as statistically significant.

## Supplementary Material

Supplementary figures and tables.

## Abbreviations

HEK293-hTau: HEK293 cells with stable expression of wild-type full-length human tau
AD: Alzheimer's disease
PROTACs: Proteolysis Targeting Chimeras

## References

[1] Guo XD, Sun GL, Zhou TT, Wang YY, Xu X, Shi XF (2017). LX2343 alleviates cognitive impairments in AD model rats by inhibiting oxidative stress-induced neuronal apoptosis and tauopathy. Acta Pharmacol Sin.

[2] Wilson B, Geetha KM (2020). Neurotherapeutic applications of nanomedicine for treating Alzheimer's disease. J Control Release.

[3] Robinson M, Lee BY, Hanes FT (2018). Recent Progress in Alzheimer's Disease Research, Part 2: Genetics and Epidemiology. J Alzheimers Dis.

[4] Bondi MW, Edmonds EC, Salmon DP (2017). Alzheimer's Disease: Past, Present, and Future. J Int Neuropsychol Soc.

[5] Morris M, Maeda S, Vossel K, Mucke L (2011). The many faces of tau. Neuron.

[6] Ghosh AK, Osswald HL (2014). BACE1 (beta-secretase) inhibitors for the treatment of Alzheimer's disease. Chem Soc Rev.

[7] Imbimbo BP, Watling M (2019). Investigational BACE inhibitors for the treatment of Alzheimer's disease. Expert Opin Investig Drugs.

[8] Van Dyck CH (2018). Anti-Amyloid-beta Monoclonal Antibodies for Alzheimer's Disease: Pitfalls and Promise. Biol Psychiatry.

[9] Xu S, Zhang L, Brodin L (2018). Overexpression of SNX7 reduces Abeta production by enhancing lysosomal degradation of APP. Biochem Biophys Res Commun.

[10] Congdon EE, Sigurdsson EM (2018). Tau-targeting therapies for Alzheimer disease. Nat Rev Neurol.

[11] Wischik CM, Staff RT, Wischik DJ, Bentham P, Murray AD, Storey JM (2015). Tau aggregation inhibitor therapy: an exploratory phase 2 study in mild or moderate Alzheimer's disease. J Alzheimers Dis.

[12] Duggal P, Mehan S (2019). Neuroprotective Approach of Anti-Cancer Microtubule Stabilizers Against Tauopathy Associated Dementia: Current Status of Clinical and Preclinical Findings. J Alzheimers Dis Rep.

[13] Gao N, Chen YX, Zhao YF, Li YM (2017). Chemical Methods to Knock Down the Amyloid Proteins. Molecules.

[14] Konstantinidou M, Li J, Zhang B, Wang Z, Shaabani S, Ter Brake F (2019). PROTACs- a game-changing technology. Expert Opin Drug Discov.

[15] Lu M, Liu T, Jiao Q, Ji J, Tao M, Liu Y (2018). Discovery of a Keap1-dependent peptide PROTAC to knockdown Tau by ubiquitination-proteasome degradation pathway. Eur J Med Chem.

[16] Silva MC, Ferguson FM, Cai Q, Donovan KA, Nandi G, Patnaik D (2019). Targeted degradation of aberrant tau in frontotemporal dementia patient-derived neuronal cell models. Elife.

[17] Liu SJ, Zhang AH, Li HL, Wang Q, Deng HM, Netzer WJ (2003). Overactivation of glycogen synthase kinase-3 by inhibition of phosphoinositol-3 kinase and protein kinase C leads to hyperphosphorylation of tau and impairment of spatial memory. J Neurochem.

[18] Yang C, Li X, Zhang L, Li Y, Li L, Zhang L (2019). Cornel iridoid glycoside induces autophagy to protect against tau oligomer neurotoxicity induced by the activation of glycogen synthase kinase-3beta. J Nat Med.

[19] Huang HJ, Chen SL, Hsieh-Li HM (2015). Administration of NaHS Attenuates Footshock-Induced Pathologies and Emotional and Cognitive Dysfunction in Triple Transgenic Alzheimer's Mice. Front Behav Neurosci.

[20] Sanguinetti E, Guzzardi MA, Panetta D, Tripodi M, De Sena V, Quaglierini M (2019). Combined Effect of Fatty Diet and Cognitive Decline on Brain Metabolism, Food Intake, Body Weight, and Counteraction by Intranasal Insulin Therapy in 3xTg Mice. Front Cell Neurosci.

[21] Ma QL, Zuo X, Yang F, Ubeda OJ, Gant DJ, Alaverdyan M (2013). Curcumin suppresses soluble tau dimers and corrects molecular chaperone, synaptic, and behavioral deficits in aged human tau transgenic mice. J Biol Chem.

[22] Sengupta U, Guerrero-Munoz MJ, Castillo-Carranza DL, Lasagna-Reeves CA, Gerson JE, Paulucci-Holthauzen AA (2015). Pathological interface between oligomeric alpha-synuclein and tau in synucleinopathies. Biol Psychiatry.

[23] Arendt T, Stieler JT, Holzer M (2016). Tau and tauopathies. Brain Res Bull.

[24] Kovacs GG (2017). Tauopathies. Handb Clin Neurol.

[25] Jin J, Wu Y, Chen J, Shen Y, Zhang L, Zhang H (2020). The peptide PROTAC modality: a novel strategy for targeted protein ubiquitination. Theranostics.

[26] Li X, Lei P, Tuo Q, Ayton S, Li QX, Moon S (2015). Enduring Elevations of Hippocampal Amyloid Precursor Protein and Iron Are Features of beta-Amyloid Toxicity and Are Mediated by Tau. Neurotherapeutics.

[27] Roberson ED, Halabisky B, Yoo JW, Yao J, Chin J, Yan F (2011). Amyloid-beta/Fyn-induced synaptic, network, and cognitive impairments depend on tau levels in multiple mouse models of Alzheimer's disease. J Neurosci.

[28] Biundo F, Del Prete D, Zhang H, Arancio O, D'Adamio L (2018). A role for tau in learning, memory and synaptic plasticity. Sci Rep.

[29] Roberson ED, Scearce-Levie K, Palop JJ, Yan F, Cheng IH, Wu T (2007). Reducing endogenous tau ameliorates amyloid beta-induced deficits in an Alzheimer's disease mouse model. Science.

[30] Tai HC, Schuman EM (2008). Ubiquitin, the proteasome and protein degradation in neuronal function and dysfunction. Nat Rev Neurosci.

[31] Guo JL, Buist A, Soares A, Callaerts K, Calafate S, Stevenaert F (2016). The Dynamics and Turnover of Tau Aggregates in Cultured Cells: INSIGHTS INTO THERAPIES FOR TAUOPATHIES. J Biol Chem.

[32] Lee MJ, Lee JH, Rubinsztein DC (2013). Tau degradation: the ubiquitin-proteasome system versus the autophagy-lysosome system. Prog Neurobiol.

[33] Fa M, Puzzo D, Piacentini R, Staniszewski A, Zhang H, Baltrons MA (2016). Extracellular Tau Oligomers Produce An Immediate Impairment of LTP and Memory. Sci Rep.

[34] Lasagna-Reeves CA, Castillo-Carranza DL, Sengupta U, Clos AL, Jackson GR, Kayed R (2011). Tau oligomers impair memory and induce synaptic and mitochondrial dysfunction in wild-type mice. Mol Neurodegener.

[35] Soria Lopez JA, Gonzalez HM, Leger GC (2019). Alzheimer's disease. Handb Clin Neurol.

[36] Sydow A, Van der Jeugd A, Zheng F, Ahmed T, Balschun D, Petrova O (2011). Tau-induced defects in synaptic plasticity, learning, and memory are reversible in transgenic mice after switching off the toxic Tau mutant. J Neurosci.

[37] Sato C, Barthelemy NR, Mawuenyega KG, Patterson BW, Gordon BA, Jockel-Balsarotti J (2018). Tau Kinetics in Neurons and the Human Central Nervous System. Neuron.

[38] Wu Q, Chen Y, Cui G, Cheng Y (2009). Wortmannin inhibits K562 leukemic cells by regulating PI3k/Akt channel *in vitro*. J Huazhong Univ Sci Technolog Med Sci.

[39] Toullec D, Pianetti P, Coste H, Bellevergue P, Grand-Perret T, Ajakane M (1991). The bisindolylmaleimide GF 109203X is a potent and selective inhibitor of protein kinase C. J Biol Chem.

