# Supplementary material for: A novel small-molecule PROTAC selectively promotes tau clearance to improve cognitive functions in Alzheimer-like models
Source: Theranostics. 2021 Mar 11;11(11):5279–95. doi: 10.7150/thno.55680 (PMC8039949; doi:10.7150/thno.55680)
Supplement: Supplementary file 1 — Supplementary figures and tables. [file thnov11p5279s1.pdf]

Supplementary Materials

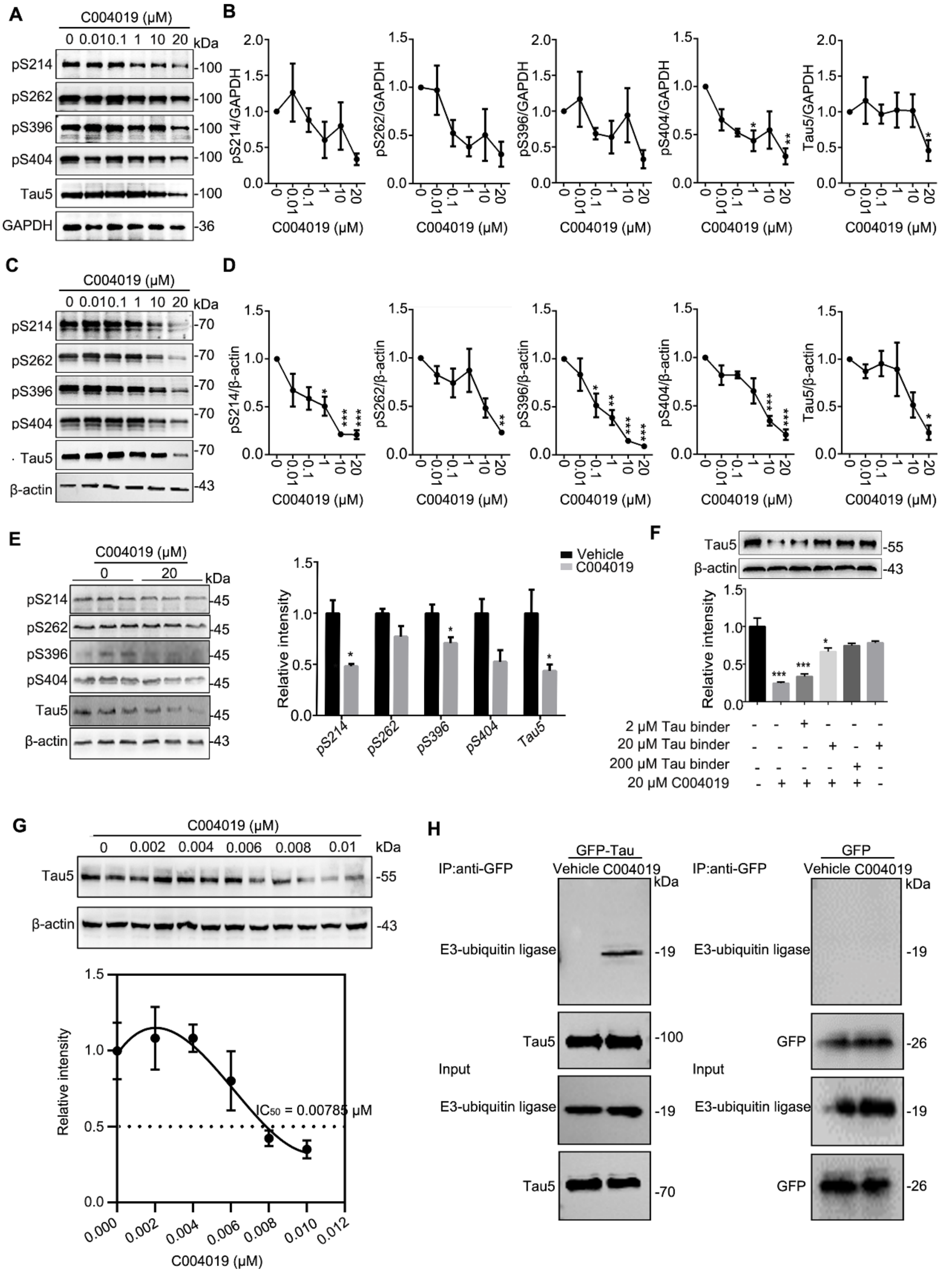

Figure S1 C004019 induces tau reduction in HEK293 cells with transient hTau expression in E3-ubiquitin-dependent manner.

(**A-D**) HEK293 cells with transient transfection of EGFP-hTau (A-B) and

3xFlag-hTau (C-D) were incubated with C004019 of different concentrations (0, 0.01, 0.1, 1, 10, 20 μM) for 24 h, and a concentration-dependent tau reduction was detected by Western blotting.

(**E**) C004019 (20 μM) decreased phosphorylated tau in SH-SY5Y cells measured by Western blotting.

(**F**) Tau binder at high concentrations (20 μM and 200 μM) blocked the effect of C004019 in removing tau proteins in HEK293-hTau cells treated with different concentrations of tau binder (2 μM, 20 μM, and 200 μM) and 20 μM C004019 measured by Western blotting.

(**G**) HEK293-hTau cells were incubated with C004019 of different lower concentrations (0, 0.002, 0.004, 0.006, 0.008, 0.01 μM) for 24 h, and a lower concentration-dependent tau reduction was detected by Western blotting, IC_{50} = 0.00785 μM.

(**H**) C004019 induced interaction of tau with E3-ubiquitin ligase (Vhl) in EGFP-hTau cells (left) measured by immunoprecipitation using GFP antibody and Western blotting using anti-Vhl and Tau5, respectively. HEK293 transiently transfected EGFP-vector and treated with C004019 was used as a negative control (right).

Data were expressed as mean ± SEM, (B, D, E, F) *P < 0.05, **P < 0.01, ***P < 0.001 vs. 0 μM. Data in (B, D, F, G) were analyzed by one-way ANOVA. Data in (E) were analyzed by Student's t-test.

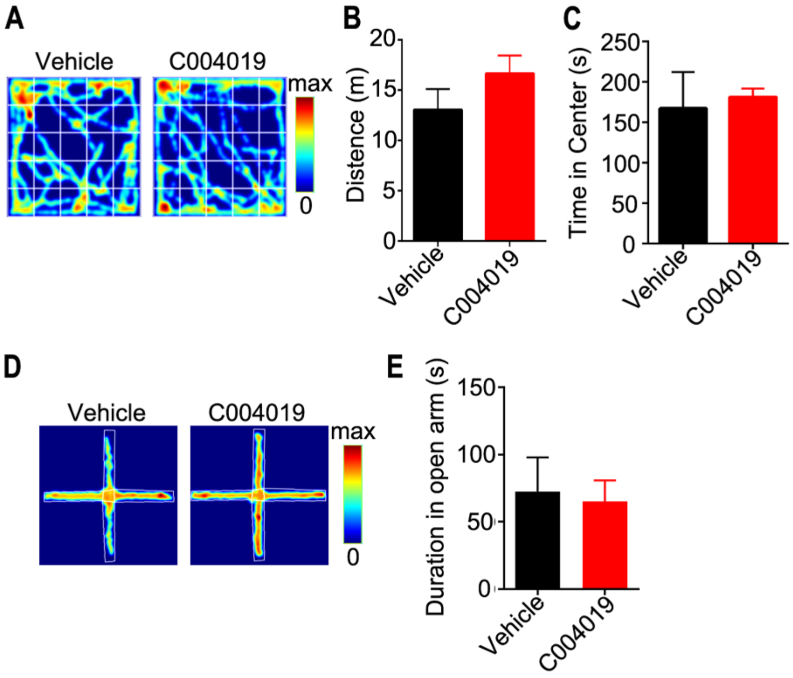

Figure S2 C004019 did not affect motor ability and anxiety level of hTau mice.

(**A-C**) C004019 treatment did not change the movement distance and the time spent staying in center during open field test (OFT) in hTau transgenic mice.

(**D-E**) C004019 did not affect open arm duration in elevated plus maze (EPM) test.

Data were expressed as mean ± SEM (n = 5 ~ 6 for each group), Data were analyzed by Student's t-test.

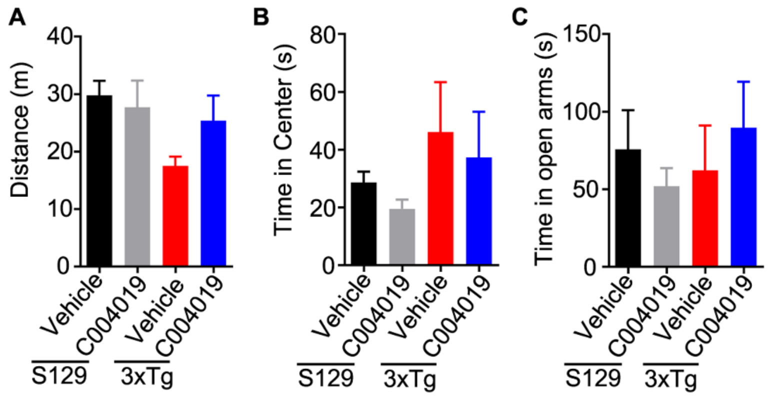

Figure S3 C004019 did not affect motor ability and anxiety level of 3xTg-AD mice.

(**A-B**) C004019 did not affect movement distance and center duration in OFT.

(**C**) C004019 did not affect the open arm duration in EPM test.

Data were expressed as mean ± SEM (n = 9 ~ 10 for each group). Data were analyzed by one-way ANOVA.
